# Sourdough Yeast Strains Exhibit Thermal Tolerance, High Fermentative Performance, and a Distinctive Aromatic Profile in Beer Wort

**DOI:** 10.3390/foods13071059

**Published:** 2024-03-29

**Authors:** Isabel E. Sánchez-Adriá, Gemma Sanmartín, Jose A. Prieto, Francisco Estruch, Francisca Randez-Gil

**Affiliations:** 1 Department of Biotechnology, Instituto de Agroquímica y Tecnología de los Alimentos, Consejo Superior de Investigaciones Científicas, Avda. Agustín Escardino, 7, 46980 Paterna (Valencia), Spainprieto@iata.csic.es (J.A.P.); 2 Department of Biochemistry and Molecular Biology, Universitat de València, Dr. Moliner 50, 46100 Burjassot (Valencia), Spain; francisco.estruch@uv.es

**Keywords:** brewing, *Saccharomyces cerevisiae*, stress tolerance, warm pitching, volatile compounds

## Abstract

The increasing popularity of home brewing and the fast evolution of craft beer companies have fuelled the interest in novel yeasts as the main actors diversifying the beer portfolio. Here, we have characterized the thermal tolerance and brewing-related features of two sourdough (SD) isolates of *Saccharomyces cerevisiae*, SDy01 and SDy02, at different temperatures, 20 and 37 °C, comparing them with commercial brew strains, AaB and kNB. The SD strains exhibited tolerance to the main brewing-related stress conditions and increased growth rates and lower lag phases than the reference beer strains at both temperatures. Consistent with this, SDy01 and SDy02 displayed higher fermentative activity in terms of sugar rate depletion and the release of metabolic by-products. Moreover, SDy01 and SDy02 brewing at 20 °C increased their total amount of volatile compounds (VOCs), in particular, their esters and carboxyl compounds, as compared to the reference AaB strain. In contrast, fermentation at 37 °C resulted in a drastic reduction in the number of VOCs in wort fermented with SD yeast, especially in its level of esters. In conclusion, our results stress the high fermentative performance of SD strains in beer wort and their ability to provide a complex and specific aromatic profile at a wide range of temperatures.

## 1. Introduction

The microbial ecosystem of natural sourdoughs encompasses a mix of lactic acid bacteria (LAB) and yeast able to face a stressful environment characterized by a high content of weak acids in a microaerophilic matrix, where maltose predominates as a carbon source. These specific conditions limit microbial diversity, which often is reduced to one or two species of LAB and yeast, with *Kazachstania humillis* and *Saccharomyces cerevisiae* being the most frequently found baking yeasts [1]. Nevertheless, sourdough of different origins exhibits large strain diversity as its microbial composition depends on the bakery environment, ingredients and process conditions [2,3]. In addition, the microbial heterogeneity across diverse sourdoughs is the result of continuous backslopping [4], which offers us the opportunity to compose genotypic diversity and fix the best-adapted evolved variants in mature sourdough.

The strain diversity of sourdough baker’s yeast might be used to improve traditional fermentations closely related to baking. Indeed, most baker’s yeast strains share a traditional common use and domestication trajectory with beer strains, which has been evidenced by large-scale phenotyping and genotyping [5]. An analysis of hundreds of industrial strains of *S. cerevisiae* has demonstrated that most baker’s yeast strains are grouped in a mixed clade that is shared with some beer strains [6,7]. This is somewhat expected given that human-driven selection toward features suited to distinct niches is the main source of genetic and phenotypic variation amongst *S. cerevisiae* lineages [8]. For example, many genes involved in maltose metabolism, the main carbon source of cereal-based fermentations, are amplified in beer, sake and bread yeast populations [8,9]. Similarly, osmotolerance genes are overrepresented in both baking and brewing yeasts [6]. Nevertheless, *S. cerevisiae* strains display genomic and metabolic peculiarities that differ depending on their technological use [5]. Indeed, there is evidence of the emergence of origin-dependent properties in these strains, which are the result of a phenotypic evolution driven by environmental constraints [10]. The selective pressure exerted on bakers’ strains in the sourdough environment, characterised by a low pH, high acidity and reduced water activity, is totally different from that on wine, brewers’ or distillers’ yeast [1,2,3]. In addition, sourdough yeast strains are characterised by a high CO_2_ production rate, a property which is needed for bread-making [11] but detrimental to by-product formation. There is also evidence that the metabolic features common to sourdough strains include a high production of esters among their main volatile organic compounds [1]. Hence, baker’s yeast strains show common and distinct metabolic characteristics to those of brewing strains, and consequently offer the opportunity for innovation and differentiated finished products.

A rising development in food microbiology involves employing microbial cross-over fermentation. This technological approach utilizes microorganisms commonly employed in certain fermentation processes to enhance the quality of other agri-food productions/chains [12,13]. Indeed, several studies have reported that *S. cerevisiae* baker’s yeast strains can be employed to ferment brewer’s wort [7,14,15,16,17] and are typically used to brew common Eastern European beers like kvass [18] and Finland’s sahti [19,20]. Sourdough cultures are also interesting reservoirs of maltose-negative yeasts for low-alcohol beer brewing [21], fruit beers [22] and craft beers with potential healthy added value [23,24,25,26]. Beers obtained using sourdough strains are also characterized by aromatic differences that are in consonance with their specific metabolic features, which appear to promote the synthesis of volatile compounds [14], in particular esters [1], instead of the fuse alcohols more abundant in brewing yeasts [27]. Although this is an important trait, other properties such as short lag phase, high fermentation rate or broad temperature tolerance were secondary or not considered in previous studies. The latter is especially important as there is a growing interest in brewing at temperatures of 35–40 °C, which are well above those typically used for commercial ales [28]. Warmer fermentations result in shorter lag phases and fermentation times, which reduces the risk of contamination, increases the economy of the process and saves cooling costs, particularly in geographical locations such as Southern Europe. Moreover, brewing at high temperatures appears to have no undesirable effects on the product’s sensory properties and even reduces off-flavours. For example, wort pitching with traditional Norwegian kveik ale thermotolerant yeasts produces clean and fresh-tasting beers [28,29,30].

The increasing demand for novel beer styles, the emergence of the craft brewing community and the limited number of beer strains used in industrial production [31] have stimulated interest in exploring novel sources of yeast strains with potential uses in brewing [32,33]. In addition, the utilization of novel yeast strains which are better adapted to a wide range of temperatures could significantly enhance brewing sustainability in this era of climate change. Here, we have evaluated the brewing potential of two sourdough strains of *S. cerevisiae*, SDy01 and SDy02, which were isolated in a bioprospecting study of the microbiota of industrial sourdough [1], by comparing them with commercial brewing strains typically used for conventional and warm pitching. The sourdough isolates of *S. cerevisiae* display high metabolic activity in media containing maltose as the main sugar, a tolerance to acid, ethanol and oxidative stress, and differential aromatic characteristics, particularly the presence of esters as the major VOC family [1]. Hence, we explained how the use of these baker’s yeast strains could be a promising alternative to improve brewing’s technological parameters across a wide range of temperatures and endow the final beer with a specific and complex aromatic character.

## 2. Materials and Methods

### 2.1. Strains, Media and Culture Conditions

Throughout this work, two *S. cerevisiae* strains, SDy01 and SDy02, isolated from sourdough in our laboratory [1], were examined. As a reference, two commercial brewing yeasts provided by the Tyris Brew Co. (Paterna, Valencia) were utilized: an American West Coast Ale Yeast, named AaB (American ales Brew), and a kveik Norwegian farmhouse yeast, named kNB (kveik Norwegian Brew). Both were classified as *S. cerevisiae* top fermenting yeasts.

Previously described standard methods were followed for media preparation [34]. Yeast cells were propagated from cryo-cultures in solid YPD (2% glucose, 2% peptone, 1% yeast extract and 2% agar) at 30 °C. Ale malt wort with an extract content of 10 Plato (10°P) was prepared by dissolving 131.8 g of hop-less Pilsen Light liquid malt extract, LME, (Briess Malt & Ingredients, Co., Chilton, WI, USA) in distilled water to a total volume of 1 L. The suspension was centrifuged at 3000 rpm for 10 min/4 °C and the clarified wort was sterilized by autoclaving at 110 °C for 10 min [35]. Finally, the obtained wort was chilled and stored at 4 °C until use.

### 2.2. Yeast Viability

For the viability evaluation, 10-fold serial dilutions of each sample were made, and 100 μL of different dilutions was plated in triplicate on solid YPD medium. The plates were incubated at 25 °C for 48–72 h, and colonies were enumerated manually. Only dilutions containing 30–300 colonies on each plate were considered. The number of viable cells is expressed as a mean (±SD) of the log_10_-transformed number of colony-forming units (cfu) per ml of wort.

### 2.3. Stress Experiments

The effect on yeast growth of brewing-related stress conditions was assayed by a drop test. Cells were grown to their mid-exponential phase at 25 °C (OD_600_~0.8). Then, 10-fold serial dilutions (10^−1^–10^−3^) were prepared and 3 μL aliquots of each dilution were applied over solid YPD lacking or containing 1.2 M sorbitol, 200 mM lactic acid (pH 3.8), 10% ethanol (*v*/*v*), or 1 g/L of iso-α acids (Hop-extract pre-isomerized, 6%, Browland, Belgium). Temperature trials at 15, 25, 37 and 40 °C, and a multi-stressor condition consisting of YPD medium supplemented with 10% ethanol, 200 mM lactic acid and 0.25 g/L of iso-α acids was also tested. Colony growth was inspected after 2–6 days at 25 °C or at the indicated temperature.

### 2.4. Growth Kinetics

Yeast strains were grown overnight in wort medium at 25–30 °C and 180 rpm, in biological triplicates. Then, aliquots of their cultures were inoculated to a final volume of 200 μL in 96-well microplates (initial OD_600_~0.1). At least 4 technical replicates of each culture were analysed. Specific wells were run without inoculation for background correction. The plates were then sealed and incubated at 20 or 37 °C under static conditions using a SPECTROstar analyser (OMEGA) inside an incubator (EQUITEC), with temperature and humidity (70%) controlled. Growth was automatically recorded by measuring their OD_600_ at 2 h intervals during a 48 h period, with vigorous shaking before each read. The observed blank in each run was subtracted from the measured OD_600_ values, and the resulting data (observed OD_600_; OD_obs_) were then converted to corrected OD_600_ (OD_cor_) according to the formula OD_cor_ = OD_obs_ + 0.449(OD_obs_)^2^ + 0.191(OD_obs_)^3^ [36]. Relevant growth parameters, their specific growth rate constant *μ*_max_ (the slope of the linear portion), the generation time *g* (equal to 0.301/slope), the division rate *v* (equals to 1/*g*) and the lag time *λ* (the interception between the slope and a straight line corresponding to the initial OD_cor_), were calculated from the curve of the logarithmically transformed OD_cor_ data, which was presented as a function of time. The maximal OD_600_ (OD_max_) was calculated as the mean of the OD_cor_ measurements corresponding to the six last time points [36]. The data presented represent the average ± standard deviation (SD) of at least three curves obtained from independent cultures.

### 2.5. Small Lab-Scale Fermentations

Yeast strains were inoculated from YPD slants in wort and cultivated overnight in an orbital shaker at 180 rpm of agitation and 25 or 30 °C for low- (20 °C) and high- (37 °C) temperature fermentations, respectively. Cultures were carried out in 100 mL bottles containing 60 mL of wort inoculated with 0.3 units of OD_600_ (~3 × 10^6^ cells/mL), without shaking, at 20 °C or 37 °C. Polypropylene screw caps with gas-permeable 0.2 μm PTFE membranes (Fisher Scientific, Waltham, MA, USA) were used to ensure aeration and pressure equalisation. The fermentation progress was tracked by monitoring weight loss using a balance and was considered to be finished after 72 and 96 h for fermentation temperatures of 37 and 20 °C, respectively. After these indicated periods, the experimental beers were matured at 4 °C for 3 days. Sterile wort controls and samples taken throughout and at the end of the experiments were stored at −80 °C for further characterization. At least three biological replicates were conducted for each strain and condition.

### 2.6. Metabolite Target Analysis

Determination of the main fermentation and wort metabolites, maltotriose, maltose, glucose, fructose, glycerol, ethanol and acetic acid was carried out by High-Performance Liquid Chromatography (HPLC), using a chromatograph (Waters, Milford, MA, USA) equipped with a quaternary pump, micro vacuum degasser and refraction index and variable wavelength (PDA) detectors. Samples were filtered through 0.45 μm hydrophilic-PTFE filters and 20 μL of the samples was injected into an Ion Exclusion 6 μm column (8 × 300 mm; SH-1011, Shodex) with a guard column SH-G. Chromatographic separation was conducted under isocratic conditions with a 0.5 mL/min flow rate of 1.5 mM H_2_SO_4_ at 50 °C. The run time, including separation and column washing, was 40 min. Quantification was performed using the external standards of each compound. Calibration curves were constructed, in duplicate, with at least six points (R^2^ = 0.9959–0.9999). Values are expressed as mg/L, except for ethanol, for which the unit of measurement is percentage by volume (% *v*/*v*), and represent the mean ± SD of at least three independent experiments.

### 2.7. Volatile Organic Compounds Analysis

The volatile organic compounds (VOCs) from beer samples were isolated using the headspace solid-phase micro-extraction technique (HS-SPME) and were detected by gas chromatography–mass spectrometry (GC–MS) according to Wang et al.’s method [37], with some modifications. For each analysis, 1 mL of matured beer, previously clarified by centrifugation and filtration, was placed into a 20 mL headspace vial with 15 µL of internal standard solution (2-heptanone, Merck Life Science, Darmstadt, Germany; Rt = 9.29 min) at 42 µg/mL. The headspace was sampled by inserting a 95 μm Carboxen/Polydimethylsiloxane (CAR/PDMS) fibre (Agilent Technologies, Santa Clara, CA, USA). Using an MPS robotic autosampler (Gerstel, Mülheim an der Ruhr, Germany), the vial, previously sealed with a silicon septum, was then heated in an oven at 50 °C for 42 min and the SPME fibre was exposed for the last 30 min. When the extraction time expired, the fibre was immediately inserted into the injection port of an Agilent 7890B-5977B GC/MSD system equipped with an HP-5 capillary column (30 m × 0.25 mm × 0.25 μm) (Agilent Technologies, Santa Clara, CA, USA) for the desorption of compounds. The gas carrier was helium, with a flow rate of 0.9 mL/min, and the SPME injections were conducted in a splitless mode at 250 °C for 3 min. The temperature program was 35 °C for 3 min, increased to 160 °C at a rate of 4 °C/min and then increased to 240 °C at a rate of 10 °C/min, and then held for 2 min before a post run at 250 °C for 2 min. The temperatures of the injection port, ion source and transfer line were 250, 230 and 260 °C, respectively. To detect all the compounds, the scan mode in the *m*/*z* range of 29–400 amu (atomic mass unit) was used, with an electronic impact of 70 eV. Volatile compounds were identified and confirmed by comparison of the mass spectral data obtained with those in the NIST (National Institute of Standards and Technology) library. Quantification was performed using three external standards representative of different chemical families: hexanol (Rt = 8.84 min) for alcohols, phenylethyl alcohol (Rt = 18.20 min) for esters and 2,3-pentanodione (Rt = 3.63 min) for carboxyls and carbonyls. Calibration curves were constructed, in duplicate, with at least six points (R^2^ = 0.9867–0.9988), using commercial standards with purity levels above 99% (Merck Life Science, Darmstadt, Germany). In all cases, values are expressed as mg/L of wort and represent the mean ± SD of at least three biological replicates tested in triplicate.

### 2.8. Statistical Analysis

Sample averages were compared by a student’s *t*-test using Excel 2021 software (Microsoft Office 2021). The samples denoted with * or # were significantly different (*p* < 0.05). The comparison of more than two sets of samples was analysed by a one-way ANOVA in XlStat 2019 and values with the same letter in the same row indicate that there are no significant differences between samples (*p* <0.05), according to Tukey’s test. Normalization of the VOCs data, heatmap, correlation matrix and principal component analysis were performed using MetaboAnalyst [38] (https://www.metaboanalyst.ca/; accessed on 9 January 2024).

## 3. Results and Discussion

### 3.1. Stress Tolerance

**Figure 1:**
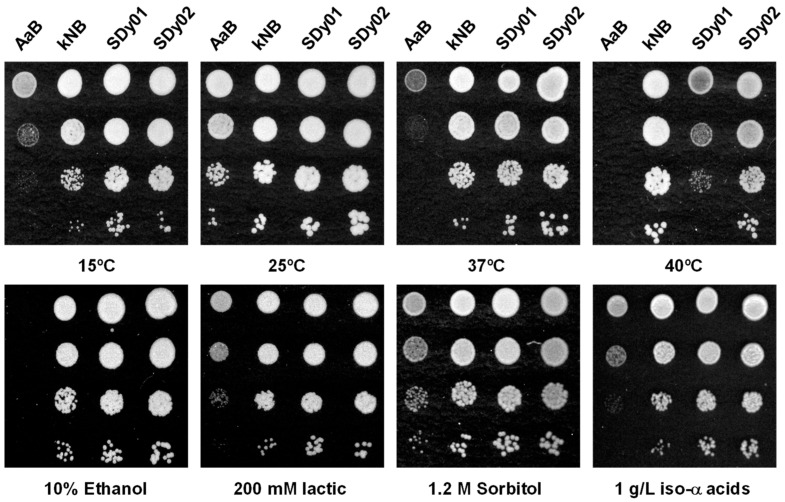
Sourdough yeast strains exhibit tolerance to typical brewing-related stress conditions. Cells of the indicated strains, AaB, kNB, SDy01 and SDy02, were assayed for their growth by a drop test. Overnight cultures grown in YPD were adjusted to an OD_600_ of 0.8. Subsequently, 10-fold serial dilutions (10^−1^–10^−3^) were prepared and 3 μL aliquots of each dilution were applied on solid YPD that was either pure or contained 1.2 M sorbitol, 200 mM lactic acid (pH 3.8), 10% ethanol (*v*/*v*), or 1 g/L of iso-α acids (pre-isomerized Hop-extract, 6%, Browland, Belgium). Colony growth was inspected after 2–6 days at 25 °C or at the indicated temperature. In all cases, a representative experiment is shown.

One of the key features that determine the selection of yeast strains for brewing is their ability to proliferate under the array of stress conditions imposed during the fermentation process. In particular, yeast can experience osmotic stress and ethanol toxicity at the onset and at the end of fermentation. A low pH, especially due to lactic acid accumulation in sour beer [39], and the presence of antimicrobial iso-α acids in hopped wort [40] are also common stressors that could impair microbial growth and metabolism. Thermal tolerance also determines the strain’s potential to be used in different brewing styles, and it is essential trait for fermentations at high temperatures. Thus, we examined the stress and thermal tolerance of two *S. cerevisiae* strains, SDy01 and SDy02, which were isolated from sourdough (SD) in our laboratory and characterised as having a high fermentative performance and resistance to typical SD stressful conditions, such as a low pH and high weak acids content [1,41]. As a control, we used two commercial brewing yeast strains typically used for conventional and warm pitching, AaB and kNB, respectively, provided by the Tyris Brew Co. The optimal temperature range for AaB is 15–22 °C, while the maximum growth for kNB varies between 35 and 40 °C. As is shown in Figure 1, the two SD strains grew as well as the kNB control in a wide range of temperatures, from 15 to 37 °C. Only at 40 °C was the growth of SDy01 limited, while the reference strain AaB was the only one not capable of growth at or above 37 °C (Figure 1). Then, we analysed their growth in the presence of 10% ethanol; a low pH (3.8), provided by 200 mM lactic acid; or 1.2 M sorbitol, as a source of osmotic stress. As can be seen, the two SD strains showed robust growth under all stress conditions, similar to that observed for the kNB reference strain (Figure 1). Neither the addition of iso-α acids at a 1 g/L concentration (Figure 1) nor a multi stressor condition combining the presence of ethanol and iso-α acids in a low-pH environment appeared to have an impact on the growth of our SD isolates. We conclude that the SD strains exhibit the required tolerance to brewing-related stress conditions.

### 3.2. Growth Parameters

Yeast growth kinetics are crucial in brewing, as even slight delays in growth after inoculation, or reduced growth rates, can compromise the fermentation’s health and efficiency, as well as the flavour, quality and economy of the final product. Considering that maltose is the primary sugar in both beer wort and bread dough, baker’s yeast strains capable of rapidly utilizing maltose would make excellent starters for beer fermentation. Consistent with this, increased specific growth rates, compared to a representative beer strain, Safbrew S-33, have been previously reported for some baker’s yeasts in experiments conducted in a synthetic YNB medium supplemented with maltose as the sole carbon source [7]. Furthermore, these baking strains displayed relatively short lag phases (*λ*) and successfully fermented hopped wort mashed at 20 °C [7], although the kinetics data on malt wort at different temperatures have not yet been reported. Therefore, we first assessed the growth constants of the two *S. cerevisiae* strains, SDy01 and SDy02, at 20 and 37 °C, the optimal fermentation temperature of the reference strains used in our study, which were AaB and kNB, respectively. Their cells were grown in microplates containing ale malt wort with 10° Plato (10°P), prepared by diluting a hop-less Pilsen-style malt barley extract. Table 1 shows the main kinetic parameters of the strains under study. As can be seen, the two SD strains showed similar behaviour in wort at 20 or 37 °C. At a low temperature, both displayed the highest growth rate and the lowest lag phase, compared with the commercial AaB strain. The cell mass yield, estimated by the highest corrected OD_600_ (OD_max_) [36], was also significantly higher for SDy01 and SDy02 (Table 1). Regarding their growth at 37 °C, the SD strains behave like the control yeast kNB, except that, again, they exhibited a significantly shorter lag phase (Table 1). We conclude that the use of SD strains in beer production has the potential to provide reduced fermentation times across a wide thermal window.

### 3.3. Fermentative Activity

**Figure 2:**
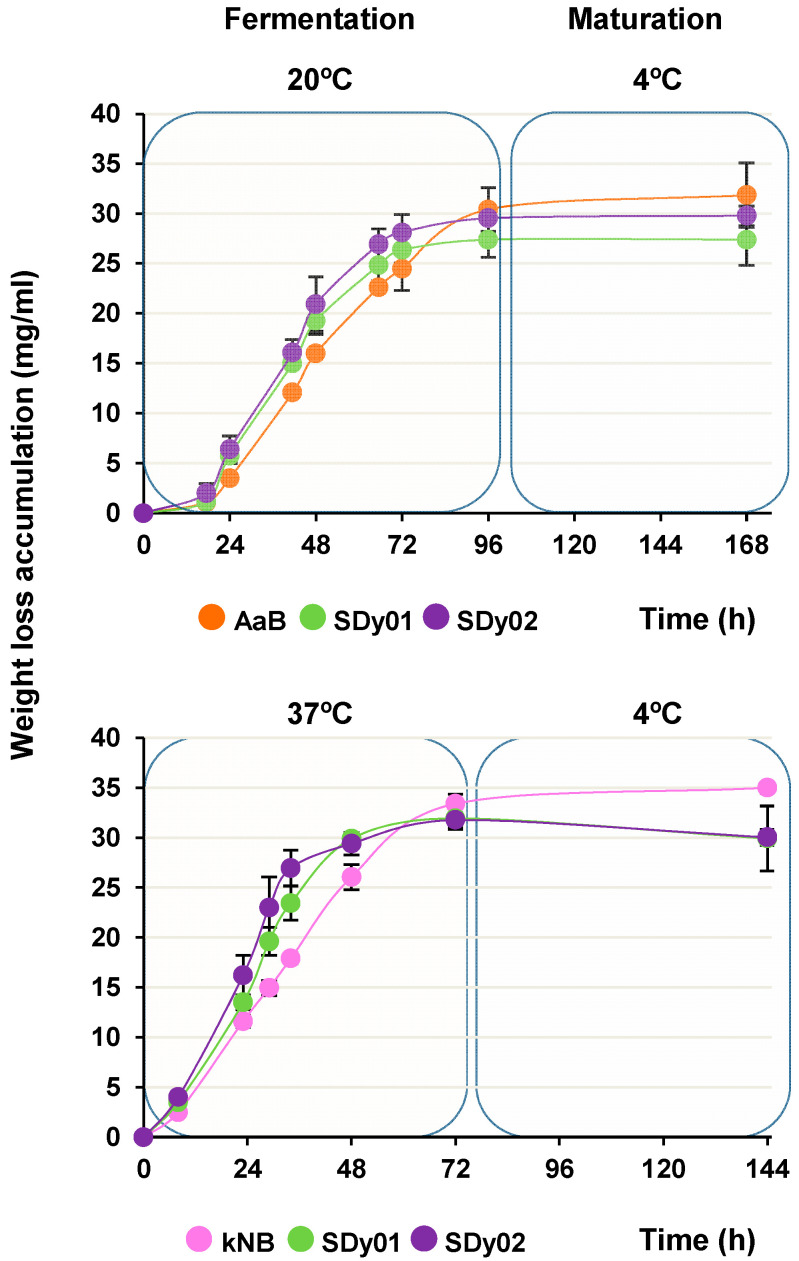
Estimation of sourdough yeast strains’ fermentative activity in wort. The graphs show the kinetics of weight loss accumulation for wort fermented using the SD strains SDy01 and SDy02 for 72 and 96 h, respectively, at 37 and 20 °C. Results for the commercial strains AaB and kNB, which served as references for fermentations at low and high temperatures, are also displayed, along with their weight loss at the end of the 96 h storage period (maturation) at 4 °C. Data represent the average ± standard deviation (SD) of at least three curves obtained from independent cultures.

We investigated the fermentative activity of the strains analysed and their capacity to transform wort into a final product with the main features of beer. Fermentations at the laboratory scale were carried out according to Thesseling et al. [35] and tracked by monitoring their weight loss, which allows for a gross evaluation of the CO_2_ released by the yeast. Figure 2 shows the profile of weight loss accumulation for wort fermented at 20 and 37 °C. The fermentation was estimated to be completed in 72 h at 37 °C and 96 h at 20 °C. The final weight loss accumulation at the end of their maturation period at 4 °C was also recorded. As can be seen, the SD strains, and in particular SDy02, displayed a faster weight loss rate compared with the control commercial strains (Figure 2). The differences were especially pronounced at 37 °C, with slope values in the straight-line portion of the curve of 0.579 ± 0.034, 0.641 ± 0.015 and 0.788 ± 0.071 * mg/mL/h for kNB, SDy01 and SDy02 (* *p* < 0.05 respect to the control). At 20 °C, SDy02 showed, again, the highest slope, 0.580 ± 0.011 * mg/mL/h (* *p* < 0.05 respect to the control), followed by SDy01 and AaB, with 0.562 ± 0.028 and 0.516 ± 0.022, respectively. Finally, there were no major differences in weight loss accumulation between the end of fermentation and their maturation period at 4 °C (Figure 2). In addition, all the yeasts tested were able to grow to maximum populations of about 7.5–8.1 and 7.3–7.7 log_10_ cfu mL^−1^ at 20 and 37 °C, respectively, from an initial inoculum level of 6.3 log_10_ cfu mL^−1^. These results are consistent with the kinetic parameters shown above, in which the SD strains displayed an increased *μ*_max_ and/or shorter *λ* (Table 1).

### 3.4. Biochemical Profile

**Figure 3:**
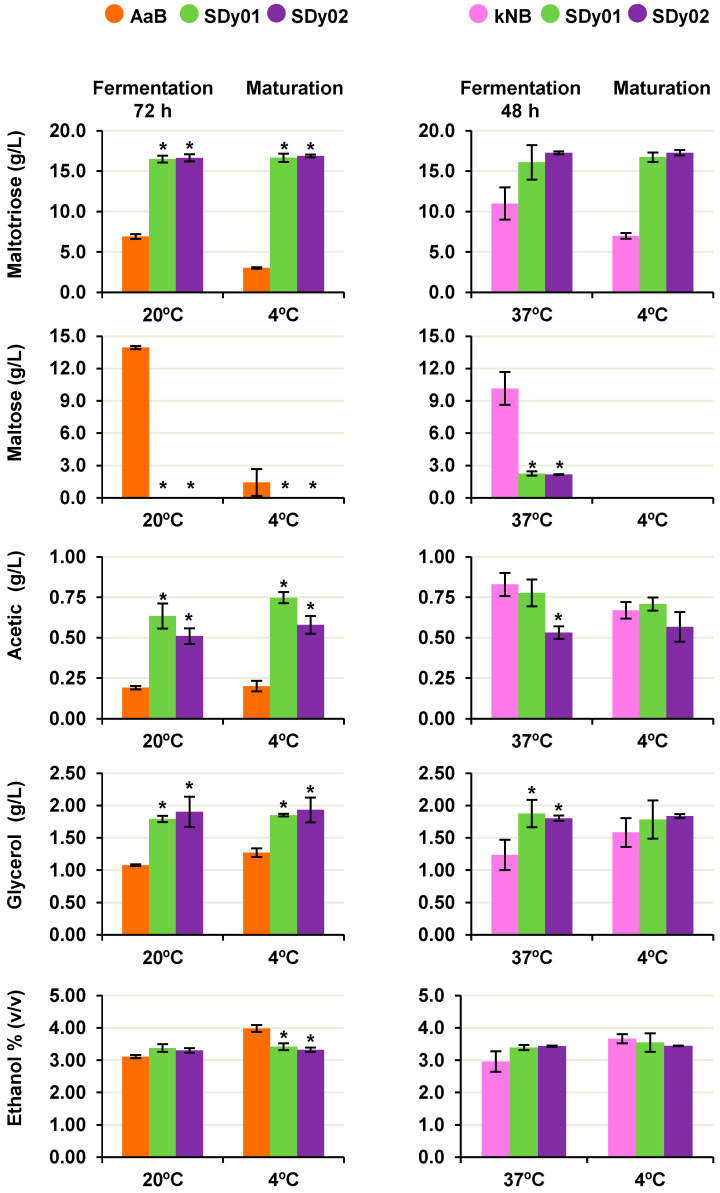
Main metabolites quantified in fermented wort samples. The content of maltotriose, maltose, glucose, fructose, glycerol, ethanol and acetic acid in wort after 48 h (37 °C) or 72 h (20 °C) of fermentation using the SD strains SDy01 and SDy02 and the commercial strains AaB and kNB, which were used as references of low- and high-temperature fermentation, is shown. Results after a 96 h maturation period at 4 °C are also shown. The concentration of each analyte was carried out by RP-HPLC, using standard calibration curves. Data represent the average ± standard deviation (SD) of at least three biological samples conducted for each strain and condition. SDy01 and SDy02 samples denoted with * were significantly different (*p* < 0.05) from their corresponding reference strain at 20 or 37 °C. Initial fermentable sugars were maltotriose: 17.95 ± 0.07 g L^−1^, maltose: 51.55 ± 0.78 g L^−1^, glucose: 10.20 ± 2.12 g L^−1^ and fructose: 3.01 ± 0.84 g L^−1^.

We then investigated whether the aforementioned results stemmed from differences in the yeasts’ rate of sugar consumption during fermentation. To this end, we analysed the concentrations of glucose, fructose, maltose, and maltotriose in the wort before the conclusion of the fermentation period. Wort samples were taken at 48 and 72 h after the onset of fermentation at 37 °C and 20 °C, respectively, and analysed using RP-HPLC. Aliquots of samples matured at 4 °C were also assayed as representative of the final product (Figure 3). As expected, glucose and fructose were not detected at the time of analysis in any of the strains under study. Hexoses, which together represented around 15% of the total sugars in the wort utilized, are the preferred substrate of *S. cerevisiae* [42]. In addition, the two SD strains achieved a full consumption of the initial maltose content regardless of the fermentation temperature, while 32% and 23% of the initial maltose were still present in wort fermented with AaB and kNB at 20 and 37 °C, respectively (Figure 3). The commercial strains also consumed maltotriose, although the extent of its utilization varied among them, as it was higher for AaB at 20 °C than for kNB at 37 °C (Figure 3), a result that could be related to the presence of specific transmembrane transporters and the strong temperature dependence of sugar transport [43]. Unlike this, SDy01 and SDy02 exhibited a low ability to utilize the trisaccharide, with no more than 8% of the initial content (Figure 3). Maltotriose utilization has been considered a trait shared between bakery and brewing yeast strains, acquired during their interaction with humans [44], although its uptake varies largely between strains [20]. Traditionally, the maltotriose-positive phenotype has been considered beneficial as its residual concentration in beer correlates inversely with the alcohol yield [20,45,46]. Nevertheless, the consumer demand for low-alcohol and non-alcohol beers is constantly increasing [47]. In addition, maltotriose-negative strains produce beer with a lower attenuation, leaving behind residual sweetness, body and mouthfeel [48].

The activity of the fermentation pathway was further evaluated by examining the by-product content of the wort before the conclusion of the fermentation period. At both 20 °C and 37 °C, the SD strains produced slightly higher amounts of ethanol than their corresponding reference strains at 72 and 48 h, respectively, but these differences were not statistically significant (Figure 3). However, wort fermented with SDy01 and SDy02 contained 66% and 76% higher glycerol concentrations, respectively, than those fermented with AaB (*p* < 0.05) at 20 °C, and 52% and 46% higher concentrations, respectively, than those fermented with kNB (*p* < 0.05) at 37 °C (Figure 3). Similarly, the acetic acid content was higher in wort fermented with SD strains at 20 °C, ranging from 0.6 to 0.7 g/L (*p* < 0.05), compared to wort prepared with AaB, which had a concentration of around 0.2 g/L. However, the acetic acid content values were similar, ranging from 0.5 to 0.7 g/L, in samples fermented at 37 °C with either kNB or the SD strains (Figure 3). Overall, the results confirm the higher fermentative activity of the SD strains, consistent with their faster consumption of maltose and release of CO_2_.

Finally, we examined the biochemical composition of the wort after its maturation period at 4 °C. During this step, the commercial strains depleted the residual maltose and reduced the content of maltotriose, with a corresponding increase in their level of glycerol and ethanol, while there was hardly change in the metabolite profile of wort fermented with SDy01 and SDy02 (Figure 3). As a result, the final contents of ethanol and other by-products in the wort fermented at 37 °C were similar between kNB and the SD strains. However, at 20 °C, the alcohol level achieved by AaB was a little higher, while its acetic acid and glycerol content remained lower (Figure 3). Once again, these results emphasize the potential of the SD strains to reduce the fermentation time at both temperatures tested, without significant changes in the beer’s main biochemical features.

### 3.5. VOCs

Samples of matured wort fermented with the strains under study were analysed by GC-MS to determine the influence of fermentation temperature and strain on their volatile profiles. In total, 30 compounds, 13 esters, 7 alcohols, 4 carboxyls, 2 carbonyls and 4 compounds belonging to other families, were identified and quantified (Table 2). As is shown, the use of SD strains had a great influence on the aromatic profile of wort fermented at 20 °C. Remarkably, SDy01 and SDy02 increased the total amount of VOCs present in wort by 1.7 and 1.8 times compared to the reference AaB strain, respectively. This increase was extended to all functional groups, but the change was not significant for alcohols and carbonyls in the SDy01 samples. As a result, esters, which confer a fruity–flowery aroma to beer [49], were the dominant chemical group in wort fermented with both SD strains, covering 50–44% of the total VOCs (Table 2). Interestingly enough, these VOCs are highly flavour-active compounds in beer [50] and variations in their concentration and proportion contribute to a beer’s specific ‘bouquet’ [51]. The differences were notable for the most abundant molecular species, acetate esters like 2-phenyl ethyl acetate (roses, honey) and 1-butanol-3-methyl acetate (banana and pear) and fatty acid esters like ethyl octanoate (sour apple), ethyl decanoate (brandy, grape, pear) and ethyl 9-decenoate (fruity, fatty). Unlike this, alcohols predominated, as could be expected, in the AaB wort, at 69% of its VOCs (Table 2). The higher percentage of alcohols accounts for the highest absolute concentrations in wort fermented with most brewing yeasts, such as ale or lager [50], and enhance the flavour of beer by adding alcoholic perception and warm mouthfeel [52]. Phenyl ethanol (rose, floral) and 3-methyl-1-butanol (fruity, sweet) were the most abundant alcohol species in all samples and their content did not differ too significantly between strains. These are commonly found in beers and are involved in the aroma profile of those fermented with *Saccharomyces* strains [53]. In contrast, minor higher alcohols, like 1-heptanol, were produced by AaB, while 1-octanol and 2-decen-1-ol were significantly more abundant in the samples fermented with the SD strains (Table 2).

The higher level of esters in wort fermented with SD yeast suggests that the synthesis of medium-chain fatty acids, which are the precursors and the limit factor for fatty acid esters’ formation [54,55], is much higher in SDy01 and SDy02 than in the brewing yeast AaB under these conditions. Consistent with this, the total amount of medium-chain fatty acids, including octanoic, 9-decenoic and decanoic acid, increased 9 and 12 times in SDy01 and SDy02, compared to the reference strain, respectively (Table 2). Regarding other compounds, the two SD strains displayed a positive “phenolic-off-flavour” (POF^+^) status [56]. Indeed, increased levels of styrene and 4-vinylguaiacol were found in wort samples fermented at 20 °C with SDy01 and SDy02 compared to those fermented with AaB, although their POF productivity was low (Table 2). These compounds are produced mainly by enzymatic decarboxylation, during fermentation, of the phenolic acids, cinnamic acid and ferulic acid, respectively [57,58,59]. Their presence in excessive concentrations, e.g., >4 mg/L, generates an undesirable clove-like aroma [60]. Nonetheless, they are essential contributors to the aromatic profile of Belgian white beers, German Weizen beers and Rauch beers, among others, and at low concentrations they also play a role in the flavour profile of many other blond and dark ale-style beers [60].

The wort fermented with kNB at 37 °C (Table 2) showed a similar profile of VOCs to AaB (at 20 °C), with alcohols as the prominent chemical group followed by esters, but with a more complex composition, characterized by the presence of a lower content of ethyl hexanoate and higher levels of carboxyls and alkanes such as tetradecane (Table 2). On the contrary, the total volatile content was drastically reduced (more than 50%) in wort fermented with SD strains at high temperatures (Table 2). The results were in some ways unexpected, as an increase in temperature for lager- (12–15 °C) or ale-type (18–25 °C) fermentations had been reported to increase the concentration of volatile compounds [61,62,63] and, in particular, esters [27]. Nevertheless, Aasen [64] found a strong decrease in esters’ formation when beer brewed with Kveik yeasts across a range of temperatures from 20 to 37 °C was tested. In line with this, fermentation at 37 °C also reduced the content of 4-vinylguaiacol to the level of the commercial kNB strain, with values that were 9 and 23% of those observed at 20 °C for SDy01 and SDy02, respectively (Table 2). This result suggests that the regulation and/or activity of the phenylacrylic acid decarboxylase (Pad1) of SD yeast strains, which transform ferulic acid in 4-vinylguaiacol [65], could be temperature-sensitive. Altogether, our results stress the ability of SD strains to provide aroma diversity across a wide range of temperatures.

**Figure 4:**
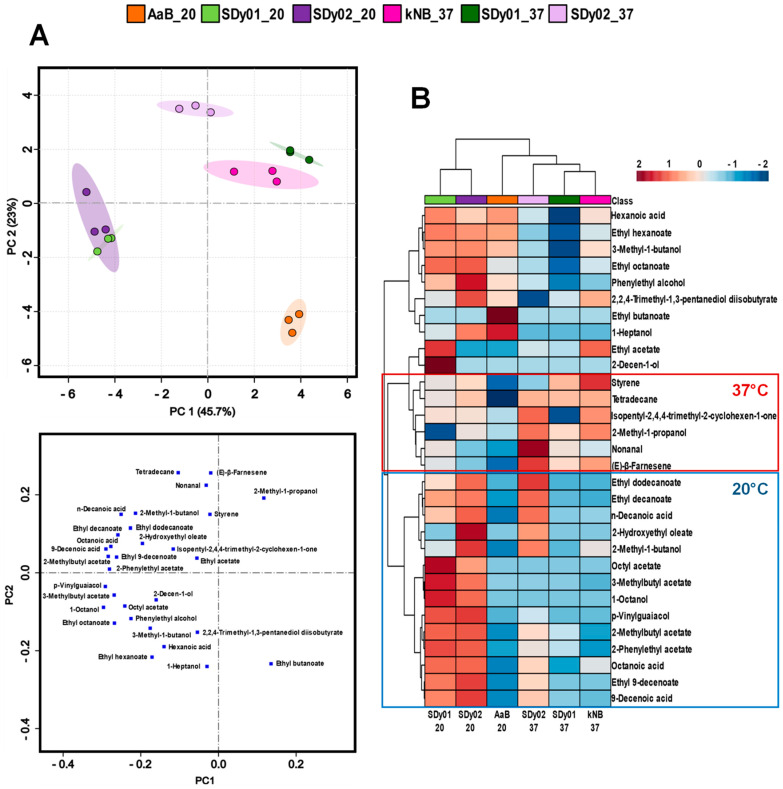
Principal component analysis (PCA) and heat map of VOCs in matured wort. (**A**) PCA of volatile compounds in matured wort, visualised using a score plot (upper panel) of the yeast used in wort fermentation: sourdough strains SDy01 and SDy02 and commercial reference strains AaB and kNB, at low (20 °C; _20) and/or high (37 °C; _37) temperatures, and loading plot of VOCs detected (lower panel). (**B**) Heatmap of VOCs in matured wort. Columns represent the different samples fermented at 20 °C or 37 °C and the rows represent the relative abundance and distribution of the 30 volatile compounds identified. Colours correspond with relative abundance values, where dark red indicates high levels and dark blue indicates low levels. At least three biological replicates were analysed.

A principal component analysis (PCA), a heat map and a correlation matrix were conducted to assess the differences in flavour production across strains and fermentation temperatures. The PCA results showed that the first two components accounted for 68.7% of the total variance observed (Figure 4). PC1 (45.7%) reflected mainly the high content of acetate and ethyl esters detected in samples fermented with the SD strains, while PC2 (23%) explained the temperature variation. As the heatmap shows (Figure 4), the hierarchical clustering grouped the wort samples fermented with SD strains at 20 °C (cluster 1) separate from the rest (cluster 2), mainly due to their higher content of 14 compounds (lower part of the heatmap, Figure 4) that represented around 37.5% of their total content of VOCs, versus the 12.5–26.4% in samples from cluster 2. On the other hand, AaB samples were placed in the positive area of PC1 (Figure 4) due to their production of ethyl butanoate as a unique volatile produced by this yeast, as reflected clearly in Figure 4 and in the correlation matrix (Figure S1). Finally, SDy02 samples were mainly placed in the negative area of PC1 due to their higher amount of 2-methylbuthyl acetate, 2-phenylethyl acetate, octanoic acid and ethyl-9-decenoate (Figure 4 and Figure S1).

## 4. Conclusions

The present work has provided evidence of the potential of *S. cerevisiae* yeast strains from sourdough to reduce brewing fermentation times over a wide range of temperatures with no apparent detriment to the beer’s main biochemical features. The improved fermentation kinetics and increased thermal tolerance of sourdough strains provide microbiological stability to the process and flexibility, as brewers can use them in typical ale-beer styles as well as in warmer-temperature brewing processes like Kvass and Finland’s sahti, among others. Our results also demonstrate the ability of sourdough strains to provide the highest overall levels of aroma compounds at 20 °C, particularly esters and acids, resulting in temperature-dependent flavour profiles. This characteristic is desirable as it allows for targeted beer fermentation to achieve a specific aroma effect.

Our results have examined the potential for brewing with sourdough yeast isolates under lab-scale conditions. Considering the number of ingredients, wort preparations and fermentation conditions used in industrial and craft brewing, additional experiments are still needed to assess the practical applicability of these sourdough strains at the brewery scale. Meanwhile, the remarkable and unique yeast strain diversity that sourdough offers can be further explored to choose the best novel strains to meet the needs of the craft beer market and consumers seeking new, distinctive flavours and experiences in beer.

## Figures and Tables

**Table 1:** Kinetic parameters of yeast strains cultured in brewing wort *.

	Temperature					
	20 °C			37 °C		
Parameter ^1^	AaB	SDy01	SDy02	kNB	SDy01	SDy02
*λ* (h)	5.93 ± 0.57 ^a^	2.86 ± 1.20 ^b^	1.04 ± 0.81 ^b^	3.65 ± 0.51 ^a^	2.63 ± 0.09 ^b^	2.31 ± 0.19 ^b^
*μ*_max_ (h^−1^)	0.068 ± 0.004 ^a^	0.082 ± 0.004 ^b^	0.080 ± 0.005 ^b^	0.261 ± 0.019 ^a^	0.262 ± 0.013 ^a^	0.266 ± 0.014 ^a^
*g* (h)	4.56 ± 0.34 ^a^	3.70 ± 0.20 ^b^	3.79 ± 0.24 ^b^	1.18 ± 0.08 ^a^	1.16 ± 0.06 ^a^	1.14 ± 0.06 ^a^
OD_max_	4.89 ± 0.10 ^a^	5.38 ± 0.16 ^b^	5.47 ± 0.20 ^b^	4.44 ± 0.54 ^a^	5.09 ± 0.24 ^a^	5.94 ± 0.50 ^b^

**^1^** Relevant growth parameters—the specific growth rate constant (*μ*_max_), the generation time (*g*), the division rate (*v*), the lag time (*λ*) and the maximal OD_600_ (OD_max_)—were calculated from the corrected OD_600_ measurements as described in the Materials and Methods section. At least three curves obtained from independent cultures of the sourdough yeast strains SDy01 and SDy02, and the commercial brewing yeasts AaB and kNB, were analysed. * Data represent the mean ± SD of at least three independent experiments (*n* = 3). Values within a row and temperature with different superscript letters are significantly different (*p* < 0.05).

**Table 2:** Concentration of VOCs in matured wort prepared with selected strains *.

	Temperature					
	20 °C			37 °C		
Compound (mg/L)	AaB	SDy01	SDy02	kNB	SDy01	SDy02
Ethyl acetate	4.9 ± 0.2 ^a^	11.1 ± 1.3 ^b^	4.9 ± 0.6 ^a^	10.0 ± 1.5 ^a^	6.2 ± 1.4 ^b#^	6.52 ± 1.08 ^b^
3-Methylbutyl acetate	14.9 ± 0.3 ^a^	52 ± 6 ^b^	38 ± 2 ^c^	12.9 ± 1.7 ^a^	15 ± 2 ^a#^	15 ± 3 ^a#^
2-Methylbutyl acetate	1.57 ± 0.12 ^a^	8.3 ± 1.6 ^b^	8.6 ± 0.6 ^b^	1.9 ± 0.4 ^a^	3.1 ± 0.4 ^ab#^	4.34 ± 0.97 ^b#^
2-Phenylethyl acetate	7.2 ± 1.7 ^a^	47 ± 7 ^b^	43 ± 8 ^b^	7 ± 2 ^a^	13 ± 3 ^b#^	16.7 ± 1.8 ^b#^
Octyl acetate	0 ± 0 ^a^	1.0 ± 0.3 ^b^	0.27 ± 0.03 ^a^	0 ± 0 ^a^	0 ± 0 ^a#^	0 ± 0 ^a#^
**TOTAL acetate esters**	28.6 ± 1.9 ^a^	119 ± 4 ^b^	94 ± 8 ^c^	32 ± 4 ^a^	36.9 ± 1.7 ^a#^	43 ± 7 ^a#^
Ethyl butanoate	0.33 ± 0.04 ^a^	0 ± 0 ^b^	0 ± 0 ^b^	0 ± 0 ^a^	0 ± 0 ^a^	0 ± 0 ^a^
Ethyl hexanoate	31 ± 3 ^a^	37.8 ± 1.3 ^a^	33 ± 5 ^a^	15 ± 2 ^a^	4.36 ± 0.09 ^b#^	11 ± 3 ^a#^
Ethyl octanoate	27 ± 5 ^a^	60 ± 7 ^b^	64.3 ± 1.6 ^b^	24 ± 5 ^a^	9 ± 2 ^b#^	21 ± 4 ^a#^
Ethyl 9-decenoate	1.3 ± 0.2 ^a^	22 ± 2 ^b^	34.9 ± 1.9 ^c^	2.87 ± 1.13 ^a^	2.8 ± 0.8 ^a#^	8.9 ± 1.6 ^b#^
Ethyl decanoate	1.0 ± 0.4 ^a^	9.8 ± 1.7 ^b^	14 ± 3 ^b^	1.5 ± 0.9 ^a^	1.4 ± 0.4 ^a#^	16 ± 2 ^b^
2-Hydroxyethyl oleate	0 ± 0 ^a^	0 ± 0 ^a^	0.55 ± 0.09 ^b^	0 ± 0 ^a^	0 ± 0 ^a^	0.14 ± 0.04 ^b#^
Ethyl dodecanoate	0 ± 0 ^a^	0.8 ± 0.2 ^a^	4.2 ± 0.7 ^b^	0 ± 0 ^a^	0 ± 0 ^a#^	7.2 ± 0.9 ^b#^
**TOTAL ethyl esters**	60 ± 8 ^a^	131 ± 9 ^b^	151 ± 3 ^c^	44 ± 9 ^a^	18 ± 4 ^b#^	64 ± 7 ^c#^
2-Methyl-1-propanol	4.8 ± 0.2 ^a^	3.2 ± 0.5 ^b^	5.4 ± 0.8 ^a^	7.0 ± 0.8 ^a^	5.7 ± 0.9 ^a#^	7.2 ± 0.3 ^a#^
3-Methyl-1-butanol	64.6 ± 1.4 ^a^	68.1 ± 1.2 ^a^	70 ± 12 ^a^	62 ± 6 ^a^	44.4 ± 1.7 ^b#^	57 ± 9 ^b^
2-Methyl-1-butanol	21.4 ± 0.8 ^a^	26.7 ± 0.4 ^a^	35 ± 4 ^b^	27 ± 3 ^a^	23.6 ± 0.9 ^a#^	32.3 ± 0.3 ^a^
1-Heptanol	1.3 ± 0.2 ^a^	0 ± 0 ^b^	1.8 ± 0.4 ^a^	0 ± 0 ^a^	0 ± 0 ^a^	0 ± 0 ^b#^
1-Octanol	0 ± 0 ^a^	1.2 ± 0.3 ^b^	0.7 ± 0.3 ^b^	0 ± 0 ^a^	0 ± 0 ^a#^	0 ± 0 ^b#^
Phenylethyl alcohol	113.4 ± 12.3 ^a^	123 ± 18 ^ab^	164 ± 26 ^b^	93 ± 15 ^a^	76 ± 14 ^a#^	102 ± 7 ^a#^
2-Decen-1-ol	0 ± 0 ^a^	0.33 ± 0.06 ^b^	0 ± 0 ^a^	0 ± 0 ^a^	0 ± 0 ^a#^	0 ± 0 ^a^
**TOTAL alcohols**	205 ± 11 ^a^	222 ± 19 ^ab^	277 ± 41 ^b^	189 ± 19 ^ab^	151 ± 15 ^b#^	198 ± 16 ^a#^
Hexanoic acid	0.89 ± 0.12 ^a^	1.03 ± 0.07 ^a^	0.6 ± 0.2 ^b^	0.5 ± 0.3 ^a^	0 ± 0 ^b#^	0 ± 0 ^b^
Octanoic acid	1.5 ± 0.3 ^a^	12.3 ± 0.5 ^b^	12.5 ± 0.9 ^b^	4.5 ± 1.6 ^a^	1.9 ± 0.3 ^b#^	6.0 ± 0.3 ^a#^
9-Decenoic acid	0.148 ± 0.013 ^a^	3.0 ± 0.4 ^b^	5.6 ± 0.3 ^c^	0.3 ± 0.2 ^a^	0.37 ± 0.05 ^a#^	1.5 ± 0.2 ^b#^
n-Decanoic acid	0.23 ± 0.03 ^a^	2.11 ± 0.16 ^b^	5.1 ± 0.7 ^c^	0.7 ± 0.3 ^a^	0.7 ± 0.3 ^a#^	4.5 ± 0.5 ^b^
**TOTAL carboxyls**	2.8 ± 0.3 ^a^	18.42 ± 1.10 ^b^	23.7 ± 1.4 ^c^	6 ± 2 ^a^	2.9 ± 0.6 ^a#^	12.30 ± 1.03 ^b#^
Nonanal	0.39 ± 0.07 ^a^	0.51 ± 0.13 ^a^	0.43 ± 0.09 ^a^	0.478 ± 0.002 ^a^	0.51 ± 0.03 ^a^	0.81 ± 0.12 ^b#^
Isopentyl-2,4,4-trimethyl-2- cyclohexen-1-one	0.116 ± 0.006 ^a^	0.20 ± 0.02 ^b^	0.19 ± 0.03 ^b^	0.46 ± 0.07 ^a^	0 ± 0 ^b#^	0.7 ± 0.2 ^a#^
2,2,4-Trimethyl-1,3- pentanediol diisobutyrate	0.41 ± 0.05 ^a^	0.287 ± 0.012 ^b^	1.52 ± 0.05 ^c^	0.7 ± 0.3 ^a^	0.27 ± 0.15 ^ab^	0 ± 0 ^b#^
**TOTAL carbonyls**	0.92 ± 0.11 ^a^	1.00 ± 0.11 ^a^	2.25 ± 0.15 ^b^	1.7 ± 0.4 ^a^	0.78 ± 0.17 ^b^	1.5 ± 0.3 ^ab#^
Styrene	0.117 ± 0.002 ^a^	0.40 ± 0.12 ^b^	0.48 ± 0.10 ^b^	1.151 ± 0.009 ^a^	0.59 ± 0.08 ^b^	0.26 ± 0.05 ^c#^
p-Vinylguaiacol	0.153 ± 0.006 ^a^	2.0 ± 0.6 ^b^	2.3 ± 0.3 ^b^	0.17 ± 0.05 ^a^	0.17 ± 0.03 ^a#^	0.33 ± 0.02 ^b#^
Tetradecane	0 ± 0 ^a^	0.6 ± 0.2 ^b^	0.80 ± 0.11 ^b^	0.9 ± 0.2 ^a^	0.84 ± 0.06 ^a#^	0.92 ± 0.12 ^a^
(E)-β-Farnesene	0.143 ± 0.013 ^a^	0.24 ± 0.06 ^a^	0.19 ± 0.02 ^a^	0.31 ± 0.08 ^a^	0.25 ± 0.03 ^a^	0.37 ± 0.03 ^a#^
**TOTAL others**	0.41 ± 0.02 ^a^	3.2 ± 0.7 ^b^	3.8 ± 0.2 ^b^	2.6 ± 0.2 ^a^	1.85 ± 0.16 ^b#^	1.9 ± 0.2 ^b#^
**TOTAL VOCs**	298 ± 20 ^a^	495 ± 31 ^b^	551 ± 51 ^b^	275 ± 34 ^ab^	211 ± 18 ^b#^	320 ± 31 ^a#^

* Data are means ± SD of at least three independent replicates (*n* = 3). Values at a given temperature and within a row showing different superscript letters are significantly different (*p* < 0.05). ^#^ indicates a significant difference between the VOCs’ values at 20 or 37 °C for SDy01 or SDy02 (*p* < 0.05).

## Data Availability

The original contributions presented in this study are included in the article; further inquiries can be directed to the corresponding author.

## Supplementary Materials

The following supporting information can be downloaded at: https://www.mdpi.com/article/10.3390/foods13071059/s1, Figure S1: Correlation plot of VOCs from maturated wort.
