# Supplementary material for: Sourdough Yeast Strains Exhibit Thermal Tolerance, High Fermentative Performance, and a Distinctive Aromatic Profile in Beer Wort
Source: Foods. 2024 Mar 29;13(7):1059. doi: 10.3390/foods13071059 (PMC11011504; doi:10.3390/foods13071059)
Supplement: Supplementary file 1 [file foods-13-01059-s001.zip › foods-2914181-supplementary/Suplementary material.pdf]

Sourdough Yeast Strains Exhibit Thermal Tolerance, High Fermentative Performance, and a Distinctive Aromatic Profile in Beer Wort

Isabel E. Sánchez-Adriá 1, Gemma Sanmartín 1, Jose A. Prieto 1, Francisco Estruch 2 and Francisca Randez-Gil 1,*

Content:

Figure S1. Correlation plot of VOCs from maturated wort.

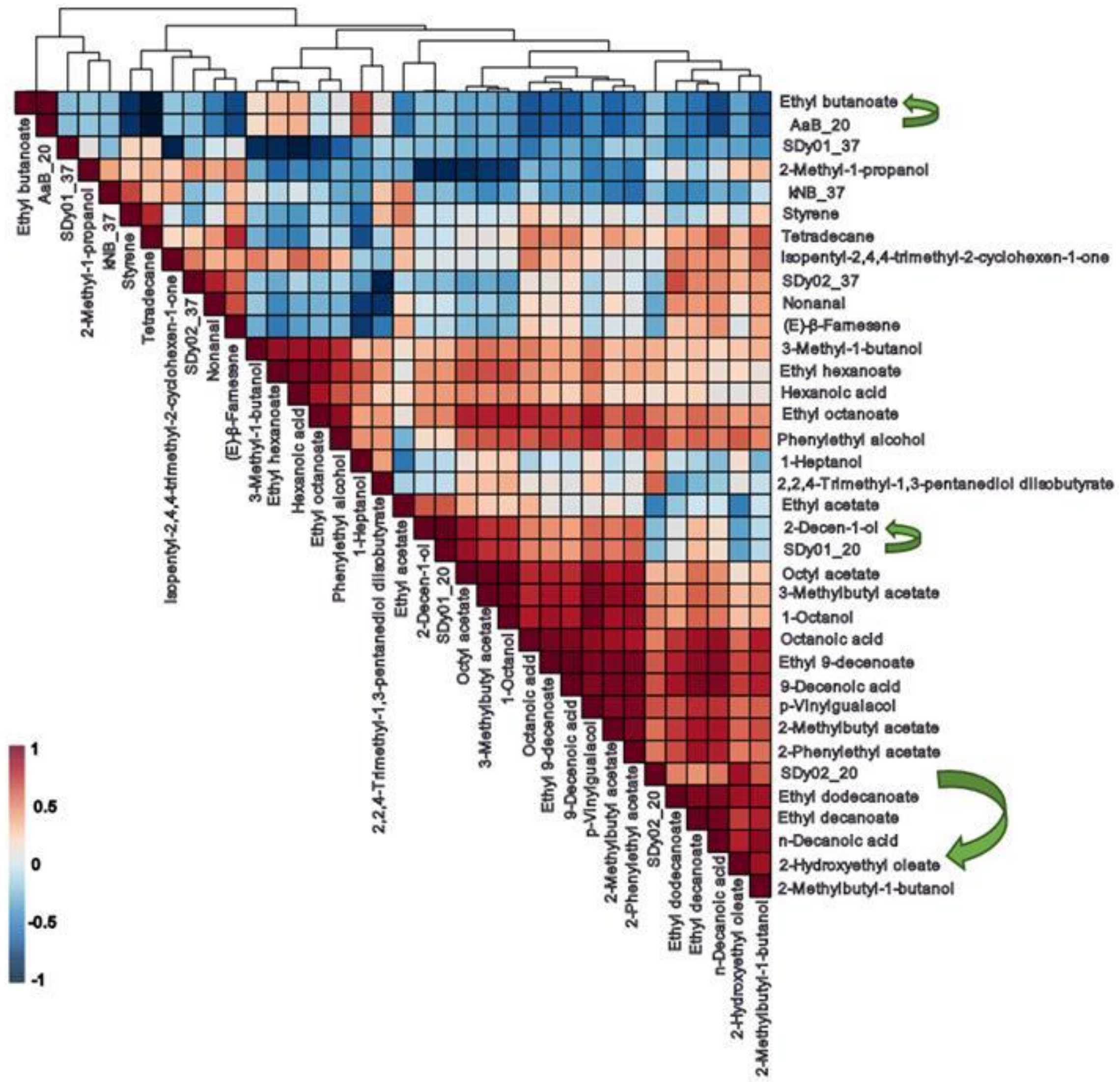

Figure S1. **Correlation plot of VOCs from maturated wort.** Pearson's correlation between pairs of yeast strains and 30 identified volatile compounds in maturated wort samples fermented by sourdough strains SDy01 and SDy02, and commercial reference strains, AaB and kNB, at low (20°C; _20) and/or high (37°C; _37) temperature. The Pearson correlation index is indicated at the scale bar, denoting the nature of the correlation with 1 for perfect positive correlation (dark red) and -1 for perfect negative correlation (dark blue). Exclusive VOCs produced by a concrete strain are denoted by a green arrow.
